# Diagnostic Performance of Serum Xenopsin-Related Peptide-1 in Gestational Diabetes Mellitus: A Prospective Observational Study

**DOI:** 10.3390/metabo16080595

**Published:** 2026-08-21

**Authors:** Yasin Karadag, Ozgur Ozdemır, Gulseren İpek Karadag, Erinç Tekin

**Affiliations:** Department of Obstetrics and Gynecology, Antalya Training and Research Hospital, University of Health Sciences, Antalya 07050, Turkey; yasin.karadag@saglik.gov.tr (Y.K.); ozgur.ozdemir73@saglik.gov.tr (O.O.); gulseren.yildirim2@saglik.gov.tr (G.İ.K.)

**Keywords:** gestational diabetes mellitus, xenopsin-related peptide-1, biomarker

## Abstract

**Background/Objectives**: Gestational diabetes mellitus (GDM) is one of the most common metabolic disorders during pregnancy and is associated with adverse maternal and neonatal outcomes. Xenopsin-Related Peptide-1 (XRP-1) has recently emerged as a potential regulator of glucose metabolism and insulin homeostasis. This study evaluated serum XRP-1 levels and assessed their diagnostic performance in women with GDM. **Methods**: In this prospective observational study, 120 pregnant women between 24 and 28 weeks of gestation were enrolled, including 60 women with GDM and 60 healthy controls. Serum XRP-1 concentrations were measured using an enzyme-linked immunosorbent assay (ELISA). Clinical, biochemical, and obstetric characteristics were compared between groups. Receiver operating characteristic (ROC) curve analysis and multivariable logistic regression were performed to evaluate the diagnostic performance and independent association of XRP-1 with GDM. **Results:** Serum XRP-1 levels were significantly higher in women with GDM than in healthy controls (3.45 ± 0.47 vs. 2.87 ± 0.46 ng/mL, *p* = 0.003). ROC analysis demonstrated moderate discriminatory performance for XRP-1 in identifying GDM (AUC = 0.658, 95% CI: 0.560–0.750), with an optimal cut-off value of 3.12 ng/mL, corresponding to 63.3% sensitivity and 61.7% specificity. Multivariable logistic regression demonstrated that serum XRP-1 remained independently associated with GDM after adjustment for maternal age, pre-pregnancy body mass index, gestational weight gain, and parity (adjusted OR 2.74, 95% CI 1.31–5.73; *p* = 0.007). HbA1c and C-reactive protein levels were also significantly higher in the GDM group. **Conclusions**: Serum XRP-1 concentrations were independently associated with GDM and demonstrated moderate diagnostic accuracy. Although XRP-1 cannot replace established diagnostic methods, it may represent a complementary biomarker for identifying gestational dysglycemia. Larger multicenter studies are required to validate its clinical utility.

## 1. Introduction

Gestational diabetes mellitus (GDM) is defined as glucose intolerance with onset or first recognition during pregnancy and represents one of the most common metabolic complications of gestation. Its global prevalence continues to rise in parallel with increasing maternal age, obesity, and sedentary lifestyles [1,2,3]. Despite advances in obstetric care, GDM remains associated with significant short- and long-term maternal and neonatal morbidity.

Maternal complications include preeclampsia, operative delivery, and future development of type 2 diabetes mellitus, while adverse fetal outcomes include macrosomia, shoulder dystocia, neonatal hypoglycemia, and increased risk of metabolic disorders later in life [4]. Improved identification of women with GDM remains an important clinical goal, particularly given the potential maternal and fetal consequences of hyperglycemia.

Gestational diabetes mellitus (GDM) is a complex metabolic disorder characterized not only by impaired glucose tolerance but also by alterations in insulin sensitivity, adipose tissue metabolism, and inflammatory pathways. Chronic low-grade inflammation has been increasingly recognized as an important component of GDM pathophysiology and may contribute to the development of insulin resistance and metabolic dysfunction during pregnancy [5]. This inflammatory milieu may also contribute to the pathogenesis of other pregnancy complications, including preeclampsia and preterm birth, which share overlapping inflammatory and metabolic pathways. Therefore, the metabolic and inflammatory dimensions of GDM should be considered together when evaluating potential biomarkers of the disease.

Currently, the oral glucose tolerance test (OGTT) is considered the gold standard for diagnosing GDM. However, the test is time-consuming, requires pre-test preparation, and may impose a logistical burden on both patients and healthcare systems. For this reason, there is ongoing interest in identifying reliable, easily measurable biomarkers that could support or complement conventional diagnostic approaches [6].

In recent years, there has been increasing interest in biomarker-based strategies for women at risk for GDM, including metabolic, inflammatory, and placental markers assessed in the first trimester. Recent scoping and narrative reviews highlight a broad range of candidate biomarkers, yet no single marker has achieved sufficient validation for routine clinical adoption [7,8,9].

Contemporary prediction efforts increasingly integrate biochemical markers with clinical risk factors to improve early risk stratification [10].

Xenopsin-Related Peptide-1 (XRP-1) is a bioactive peptide implicated in vascular permeability, inflammatory signaling, and endocrine modulation. Experimental studies have demonstrated that xenopsin administration may induce hyperglycemia, increase glucagon and cortisol secretion, and modulate insulin release [11,12,13]. Additionally, elevated circulating XRP-1 levels have been reported in women with polycystic ovary syndrome, a condition characterized by insulin resistance [14]. These findings suggest a potential role of XRP-1 in glucose metabolism and insulin regulation.

Given the central role of insulin resistance and pancreatic beta-cell dysfunction in the pathophysiology of GDM, XRP-1 may represent a biologically plausible candidate biomarker. However, its clinical relevance in gestational diabetes has not been adequately explored.

Therefore, the aim of this prospective clinical study was to evaluate serum XRP-1 levels in pregnant women with and without GDM and to determine its potential diagnostic performance and association with metabolic and inflammatory parameters.

Despite growing interest in metabolic biomarkers for gestational diabetes mellitus, data regarding Xenopsin-Related Peptide-1 in this context remain extremely limited. To date, only a small number of studies have explored circulating XRP-1 levels in insulin-resistant conditions, primarily focusing on polycystic ovary syndrome. Evidence specifically evaluating XRP-1 in gestational diabetes is scarce, and available findings are inconclusive. Therefore, there remains an unmet need to clarify whether XRP-1 is merely associated with metabolic dysregulation or whether it demonstrates clinically meaningful diagnostic performance in GDM populations.

The primary objective of this study was to determine whether serum XRP-1 levels differ between pregnant women with and without GDM and to evaluate the diagnostic performance of XRP-1 for GDM using receiver operating characteristic (ROC) curve analysis. A secondary objective was to examine the association between serum XRP-1 levels and established metabolic and inflammatory parameters. Secondary outcomes included comparisons of clinical, obstetric, and laboratory characteristics between women with and without GDM.

## 2. Materials and Methods

### 2.1. Study Design and Participants

This prospective observational study was conducted between 10 April and 10 September 2024, at the Department of Obstetrics and Gynecology, Antalya Training and Research Hospital. Ethical approval was obtained from the Institutional Ethics Committee (Approval No: 337135-44/24), and the study was conducted in accordance with the Declaration of Helsinki. All participants were informed about the purpose and procedures of the study, and written informed consent was obtained prior to enrollment and blood sample collection.

A total of 120 pregnant women were included in the study and divided into two groups: women diagnosed with gestational diabetes mellitus (GDM) (*n* = 60) and healthy pregnant controls (*n* = 60). Participants were consecutively recruited from pregnant women attending routine antenatal care between 24 and 28 weeks of gestation during the study period. Women diagnosed with GDM according to oral glucose tolerance test (OGTT) criteria were assigned to the GDM group. The control group consisted of pregnant women at comparable gestational ages who underwent routine screening and did not meet the diagnostic criteria for GDM. No randomization was performed because of the observational nature of the study. Formal matching for age, BMI, or other clinical parameters was not applied; however, both groups were evaluated within the same gestational age window and at the same institution to minimize potential selection bias.

The control group consisted of pregnant women who underwent OGTT screening at 24–28 weeks of gestation and demonstrated normal glucose tolerance according to the applicable diagnostic criteria. Control participants were not defined as complication-free throughout pregnancy; rather, they were classified as controls based on normal glucose tolerance at the time of GDM screening. Pregnancy outcomes were subsequently obtained from delivery records to evaluate obstetric and neonatal complications.

The inclusion criteria were maternal age between 18 and 35 years, singleton pregnancy, pre-pregnancy body mass index (BMI) between 18 and 35 kg/m^2^, and complete clinical records. Pre-pregnancy BMI was calculated based on self-reported preconception weight and height measured at the first antenatal visit. The upper BMI limit was selected to exclude extreme obesity, which may independently influence inflammatory and metabolic biomarkers. Age limits were applied to minimize potential confounding associated with advanced maternal age or adolescent pregnancy.

Exclusion criteria included pregestational diabetes mellitus (type 1 or type 2), endocrine disorders including adrenal diseases, androgen-secreting tumors, Cushing’s syndrome, congenital adrenal hyperplasia, and thyroid dysfunction, as well as multiple pregnancy, fetal anomalies, fetal demise, chronic systemic diseases, placental pathologies, and incomplete data.

Participants were consecutively screened during routine antenatal visits, and eligibility criteria were applied after the initial clinical assessment. Women meeting any exclusion criterion were removed prior to group allocation. Enrollment continued until 60 participants per group had been included.

### 2.2. Diagnosis of Gestational Diabetes Mellitus

GDM was diagnosed between 24 and 28 weeks of gestation using either a one-step 75-g OGTT or a two-step approach consisting of a 50-g glucose challenge test followed, when indicated, by a 100-g OGTT, in accordance with institutional practice.

Among the 60 women with GDM, 38 were diagnosed using the one-step 75-g OGTT, whereas 22 were diagnosed using the two-step 50-g glucose challenge/100-g OGTT approach. Among the 60 women in the control group, 48 had a negative 50-g glucose challenge test and therefore did not undergo the 100-g OGTT, whereas 12 had a positive 50-g glucose challenge test followed by a normal 100-g OGTT and were classified as controls.

For the 75-g OGTT, plasma glucose levels were measured after overnight fasting and at 1 and 2 h following glucose ingestion. GDM was diagnosed if at least one of the following thresholds was met or exceeded: fasting plasma glucose ≥ 92 mg/dL, 1-h glucose ≥ 180 mg/dL, or 2-h glucose ≥ 153 mg/dL.

For the two-step approach, a 50-g glucose challenge test was first performed without fasting. If the 1-h plasma glucose level was ≥140 mg/dL, a 100-g OGTT was subsequently conducted after overnight fasting. GDM was diagnosed if at least two of the following thresholds were met: fasting glucose ≥ 95 mg/dL, 1-h glucose ≥ 180 mg/dL, 2-h glucose ≥ 155 mg/dL, or 3-h glucose ≥ 140 mg/dL.

### 2.3. Clinical, Obstetric, and Laboratory Assessment

All blood samples for XRP-1 measurement were collected at the time of routine OGTT screening between 24 and 28 weeks of gestation. Thus, serum sampling was performed within a standardized gestational age window for all participants. In the control group, blood samples were obtained during the same screening visit after confirmation of normal OGTT results.

Maternal demographic, obstetric, and laboratory data were collected from hospital records. Obstetric ultrasonography was performed using the HI-VISION Avius ultrasound system (Hitachi Medical Systems, Tokyo, Japan) by a single experienced clinician to minimize inter-observer variability. Fetal biometric parameters, including biparietal diameter (BPD), abdominal circumference (AC), femur length (FL), amniotic fluid index (AFI), and estimated fetal weight (EFW), were recorded.

All laboratory parameters, including complete blood count, C-reactive protein (CRP), glucose, and HbA1c levels, were measured from blood samples obtained at the time of routine OGTT screening. Therefore, these parameters reflected metabolic and inflammatory status at the same time point as serum XRP-1 measurement.

At the time of screening venous blood samples were collected into biochemistry tubes, centrifuged at 4000 rpm for 10 min, and serum aliquots were stored at −80 °C until analysis.

HbA1c levels were measured using high-performance liquid chromatography with a Tosoh HLC-723 G8 analyzer (Tosoh Bioscience, Tokyo, Japan). Complete blood counts were analyzed using the Coulter LH-750 hematology analyzer (Beckman Coulter Inc., Brea, CA, USA). CRP and other biochemical parameters were evaluated using standard automated laboratory techniques.

### 2.4. Serum XRP-1 Measurement

Serum XRP-1 concentrations were measured using an ELISA-based assay. Synthetic Xenopsin-Related Peptide-1 (XRP-1) was used as the reference standard (Elabscience Biotechnology Co., Ltd., Wuhan, China; Catalog No. E-PP-2093). The peptide was supplied as a lyophilized powder with a reported purity of >95%. A standard curve was generated using serial dilutions of the XRP-1 standard, and serum XRP-1 concentrations were calculated from the resulting calibration curve.

The analytical measurement range was 0.05–10 ng/mL, with a sensitivity of 0.042 ng/mL. The intra-assay and inter-assay coefficients of variation were <10% and <12%, respectively.

No in vitro experiments or animal studies were performed in this research.

### 2.5. Sample Size Calculation

Sample size was calculated using G*Power version 3.1.9.7 (Heinrich-Heine-Universität Düsseldorf, Düsseldorf, Germany). Based on pilot data indicating an anticipated 10% difference in serum XRP-1 levels between groups, with a two-sided alpha level of 0.05 and 80% statistical power, a minimum of 54 participants per group was required. To compensate for potential data loss, 60 participants per group were ultimately enrolled, resulting in a total sample size of 120 participants.

### 2.6. Statistical Analysis

Statistical analyses were performed using IBM SPSS Statistics for Windows, Version 25.0 (IBM Corp., Armonk, NY, USA). Data distribution was assessed using the Shapiro–Wilk test. Continuous variables were presented as mean ± standard deviation for normally distributed data and as median (interquartile range) for non-normally distributed data. Between-group comparisons were performed using the independent-samples *t*-test or Mann–Whitney U test, as appropriate. Categorical variables were compared using the chi-square test or Fisher’s exact test.

Receiver operating characteristic (ROC) curve analysis was conducted to evaluate the discriminatory performance of serum XRP-1 levels for GDM. The optimal cut-off value was determined using the Youden Index. The area under the ROC curve (AUC), sensitivity, specificity, positive predictive value, and negative predictive value were calculated.

Univariate and multivariable logistic regression analyses were performed to assess whether serum XRP-1 was independently associated with GDM after adjustment for maternal age, pre-pregnancy BMI, gestational weight gain, and parity. Odds ratios (ORs) with corresponding 95% confidence intervals (CIs) were calculated. A two-sided *p*-value < 0.05 was considered statistically significant.

### 2.7. Ethics Statement

The study was conducted in accordance with the Declaration of Helsinki and was approved by the Ethics Committee of Antalya Training and Research Hospital (Approval No: 337135-44/24). Written informed consent was obtained from all participants before enrollment and blood sample collection.

## 3. Results

### 3.1. Outcomes

#### 3.1.1. Participant Characteristics

**Figure 1:**
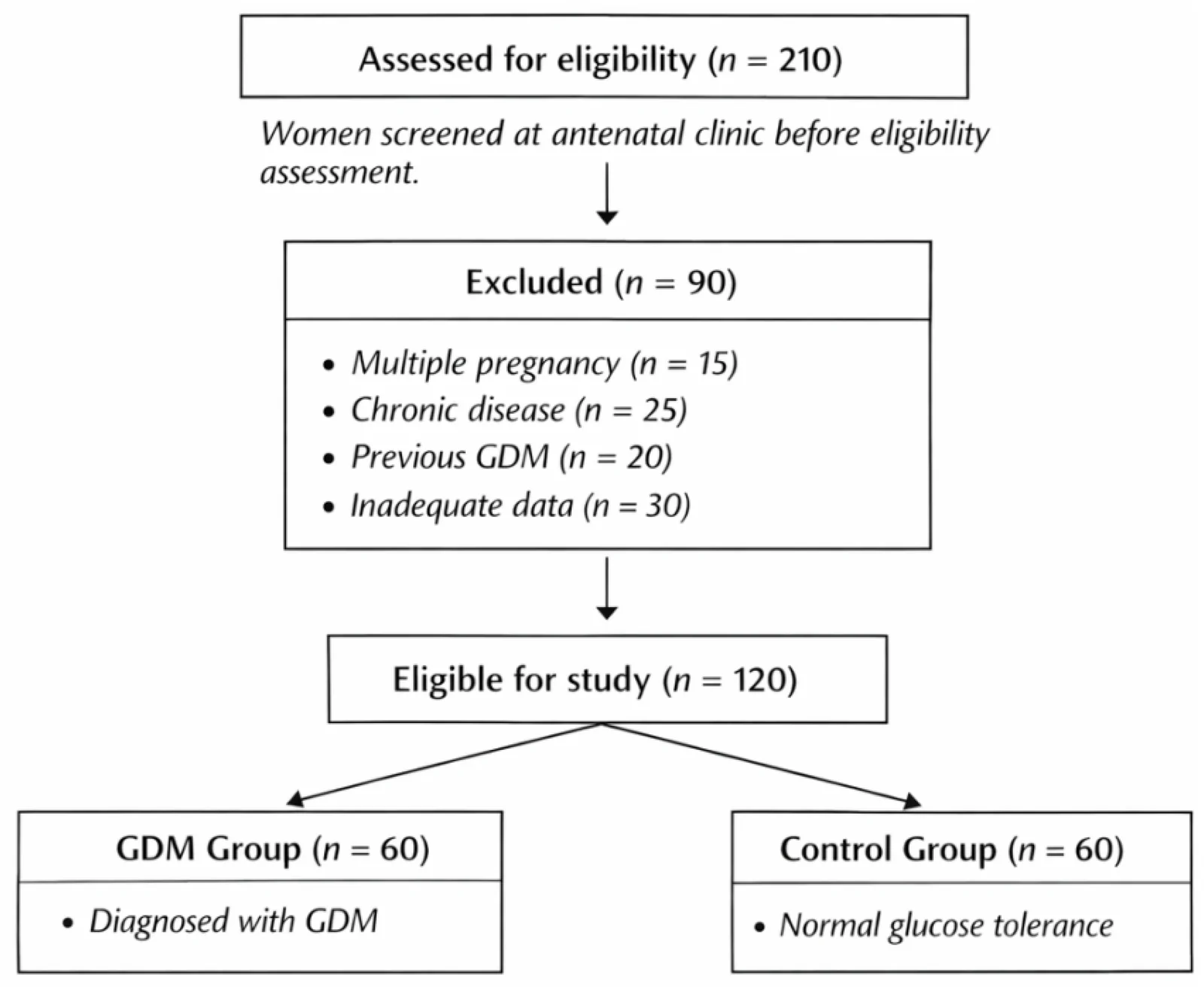
Flow diagram of participant screening, exclusion, and group allocation.

A total of 120 pregnant women were enrolled in this prospective observational study, including 60 women diagnosed with GDM and 60 healthy pregnant controls. Participant screening, exclusion, and final group allocation are presented in Figure 1.

Baseline demographic and obstetric characteristics are summarized in Table 1. Women with GDM were significantly older and had higher pre-pregnancy BMI, greater gestational weight gain, higher gravidity, and higher parity than controls (all *p* < 0.05). In addition, cesarean delivery, neonatal birth weight, and NICU admission rates were significantly higher in the GDM group. No significant differences were observed regarding fetal presentation, fetal sex, Apgar scores, curettage history, ectopic pregnancy history, or prenatal screening results.

#### 3.1.2. Glucose Metabolism and Treatment Characteristics

Glucose metabolism parameters are presented in Table 2. Fasting blood glucose and 50-g glucose challenge test values were significantly higher in women with GDM than in controls (*p* < 0.05). Among women diagnosed with GDM, 42 (70.0%) achieved glycemic control with dietary modification alone, whereas 18 (30.0%) required insulin therapy.

#### 3.1.3. Fetal Biometry

Fetal biometric findings are summarized in Table 3. Women with GDM demonstrated significantly greater biparietal diameter, abdominal circumference, femur length, and estimated fetal weight than controls (all *p* < 0.001). Amniotic fluid abnormalities were also more frequently observed in pregnancies complicated by GDM.

#### 3.1.4. Laboratory Findings

Laboratory parameters are summarized in Table 4. Compared with controls, women with GDM had significantly higher hematocrit, blood urea nitrogen, creatinine, C-reactive protein, fasting glucose, HbA1c, and serum XRP-1 concentrations (all *p* < 0.05). No significant differences were observed in hemoglobin concentration, leukocyte count, platelet count, liver enzymes, thyroid function tests, or inflammatory cell indices.

#### 3.1.5. Pregnancy Outcomes

Pregnancy-related complications observed among women with GDM are summarized in Table 5. Amniotic fluid abnormalities were the most frequent complication (26.7%), followed by preterm birth (18.3%), preeclampsia (13.3%), placenta previa (8.3%), gestational cholestasis (6.7%), and fetal growth restriction (5.0%).

#### 3.1.6. Diagnostic Performance of Serum XRP-1

**Figure 2:**
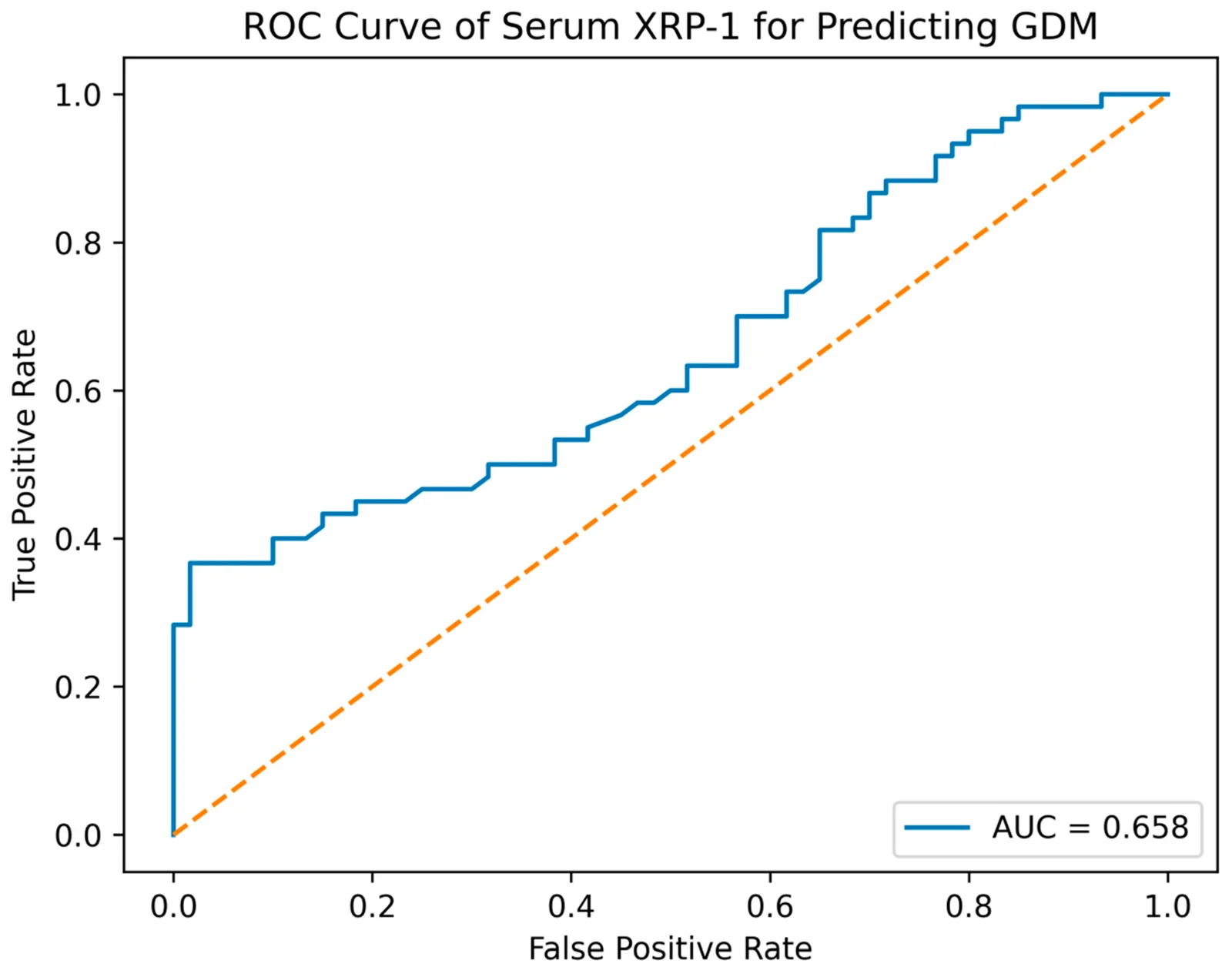
ROC Curve of Serum Xenopsin-Related Peptide-1. The orange dashed diagonal line represents the reference line for no discrimination (AUC = 0.50).

ROC curve analysis demonstrated moderate discriminatory performance of serum XRP-1 for identifying GDM, with an AUC of 0.658 (95% CI 0.560–0.750, *p* = 0.004) (Figure 2). The optimal cut-off value determined using the Youden Index was 3.12 ng/mL, corresponding to a sensitivity of 63.3%, specificity of 61.7%, positive predictive value of 62.1%, and negative predictive value of 62.9% (Table 6).

Multivariable logistic regression analysis demonstrated that serum XRP-1 remained independently associated with GDM after adjustment for maternal age, pre-pregnancy BMI, gestational weight gain, and parity (adjusted OR 2.74, 95% CI 1.31–5.73; *p* = 0.007).

**Figure 3:**
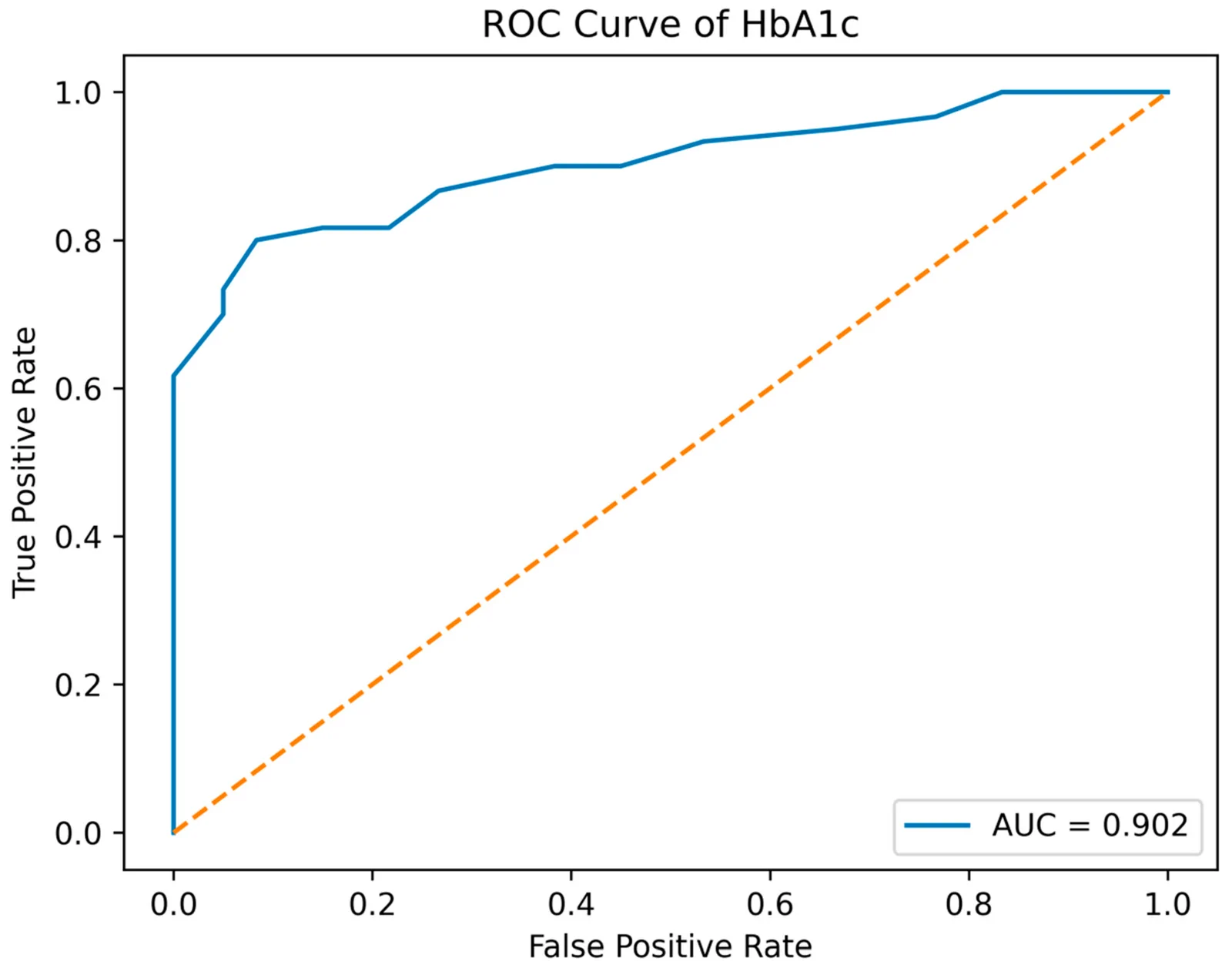
ROC Curve of HbA1c. The orange dashed diagonal line represents the reference line for no discrimination (AUC = 0.50).

For comparison, HbA1c demonstrated excellent discriminatory performance (AUC 0.902), whereas XRP-1 showed moderate diagnostic accuracy (Figure 3).

The mean between-group difference in serum XRP-1 concentration was 0.54 ng/mL (95% CI 0.21–0.87), corresponding to a moderate effect size (Cohen’s d = 0.57). Post hoc power analysis demonstrated a statistical power of approximately 84%.

## 4. Discussion

In this prospective observational study, women with gestational diabetes mellitus exhibited significantly higher circulating XRP-1 concentrations than healthy pregnant controls. Although ROC analysis demonstrated only moderate discriminatory performance, multivariable logistic regression confirmed that XRP-1 remained independently associated with GDM after adjustment for established clinical risk factors. In addition, women with GDM exhibited the expected metabolic and obstetric profile, including higher BMI, gestational weight gain, HbA1c, CRP concentrations, cesarean delivery rates, neonatal birth weight, and NICU admissions. Collectively, these findings support the potential role of XRP-1 as a complementary biomarker reflecting metabolic dysregulation in GDM rather than as a standalone diagnostic test.

Gestational diabetes mellitus is characterized by progressive insulin resistance and relative pancreatic beta-cell dysfunction. Although physiological insulin resistance increases during normal pregnancy, women who develop GDM exhibit an exaggerated metabolic response, resulting in hyperglycemia. Identifying circulating mediators involved in this process may improve understanding of disease mechanisms and assist in risk stratification [1].

Experimental studies have suggested that xenopsin-related peptides may influence glucagon and cortisol secretion, thereby potentially contributing to insulin resistance. However, glucagon and cortisol levels were not measured in the present study; therefore, the underlying mechanistic pathway cannot be confirmed in this cohort [11,12,13]. Moreover, elevated circulating XRP-1 levels have previously been reported in women with polycystic ovary syndrome, a condition strongly associated with insulin resistance [14]. These findings support the biological plausibility of XRP-1 involvement in dysglycemia.

In the present study, serum XRP-1 levels were significantly higher in the GDM group compared with controls (median 3.45 [IQR 2.98–4.12] ng/mL vs. 2.87 [2.41–3.34] ng/mL, *p* = 0.003). ROC analysis demonstrated moderate discriminatory performance (AUC 0.658). However, multivariable logistic regression confirmed that XRP-1 remained independently associated with GDM after adjustment for maternal age, BMI, gestational weight gain, and parity. Despite this association, OGTT remains the diagnostic gold standard, and XRP-1 should be considered a potential adjunct biomarker rather than a replacement test.

The additional laboratory parameters were analyzed to contextualize XRP-1 within the broader metabolic and inflammatory profile at the time of GDM screening, rather than as independent primary endpoints.

Consistent with the existing literature, women with GDM in our cohort had higher BMI, greater gestational weight gain, and increased birth weight compared with controls. Elevated HbA1c and CRP levels in the GDM group further reflect the metabolic and inflammatory milieu associated with insulin resistance. Chronic low-grade inflammation is known to contribute to impaired insulin signaling through cytokine-mediated pathways, including IL-6 and TNF-alpha activation [15,16,17]. The concurrent elevation of CRP and XRP-1 in our study may suggest a possible interaction between inflammatory and endocrine mechanisms, although causal relationships cannot be inferred from this design.

This finding is consistent with previous reports showing higher cesarean delivery rates among pregnancies complicated by gestational diabetes, particularly in the presence of suspected fetal macrosomia or labor dystocia [18].

Higher NICU admission rates in GDM pregnancies have been consistently reported and are largely attributed to neonatal hypoglycemia risk and metabolic monitoring protocols rather than severe morbidity. Therefore, the observed increase likely reflects standard neonatal precautionary care in this population.

Interestingly, hematological parameters such as leukocyte count, neutrophil-to-lymphocyte ratio, and platelet indices did not differ significantly between groups. These findings align with some previous reports but contrast with others, highlighting the heterogeneity of inflammatory markers in GDM populations [19,20,21,22,23].

Although gestational diabetes has been associated with endothelial dysfunction and enhanced platelet activation in previous studies, we did not observe a significant difference in platelet count between groups. It is important to note that platelet activation does not necessarily manifest as quantitative changes in platelet count. Rather, activation is primarily reflected by functional alterations, including changes in platelet reactivity, surface receptor expression, and mean platelet volume. Therefore, the absence of a difference in platelet count does not contradict the possibility of enhanced platelet activation in GDM. Future studies incorporating functional platelet markers may better clarify this relationship.

To date, data regarding XRP-1 in gestational diabetes are limited.

A recent prospective cohort study by Ağdemir et al. (44 GDM/44 controls) reported no significant difference in circulating XRP-1 levels between groups despite evaluating participants during the standard screening window (24–28 weeks) and diagnosing GDM using a single-step 75 g OGTT approach. In that study, the GDM group was significantly older, whereas BMI (both pre-pregnancy and at blood collection) did not differ between groups, which may have reduced the contrast in underlying insulin-resistance phenotypes. In contrast, our cohort demonstrated higher BMI and greater metabolic-inflammatory burden in the GDM group, and we observed significantly higher XRP-1 levels with good discriminatory performance. Methodological differences may also contribute, including differences in assay platforms (commercial ELISA kit details in Ağdemir et al. were limited, with measurements performed externally), and potential pre-analytical variability (sample processing/centrifugation conditions). Finally, while our larger sample size increases statistical power to detect between-group differences, the discrepant findings likely reflect a combination of population characteristics (age/BMI distribution), diagnostic pathways, and assay variability. Therefore, further multicenter studies using standardized assays and harmonized diagnostic criteria are warranted [24].

The current biomarker landscape for early GDM prediction is rapidly expanding and includes metabolic, inflammatory, placental, urinary, and genetic candidates; however, heterogeneity in study designs and limited external validation remain key barriers to clinical implementation [7,8].

Within this evolving context, exploring XRP-1 is relevant as a metabolically active peptide with plausible links to insulin resistance pathways, aligning with contemporary research trends that seek accessible blood-based biomarkers [25].

In this study, serum XRP-1 demonstrated moderate discriminatory performance for identifying GDM. Although the AUC indicates limited standalone diagnostic accuracy, multivariable logistic regression analysis confirmed that XRP-1 remained independently associated with GDM after adjustment for maternal age, BMI, gestational weight gain, and parity. These findings suggest that XRP-1 may function as a complementary biomarker rather than a replacement for OGTT. Although XRP-1 demonstrated statistically significant independent association with GDM in multivariable analysis, ROC performance indicated only moderate diagnostic accuracy.

Overall, our findings indicate that XRP-1 is independently associated with gestational diabetes mellitus and reflects the metabolic alterations accompanying the disease. Although its standalone diagnostic performance was moderate, the independent association observed after multivariable adjustment suggests that XRP-1 may complement established clinical and biochemical markers in future risk prediction models. Further prospective multicenter studies are required to validate these findings and determine whether incorporation of XRP-1 into multimarker strategies provides clinically meaningful additional diagnostic or predictive value.

The strengths of this study include its prospective design, standardized blood sampling during the recommended GDM screening window, comprehensive metabolic characterization, and the combined use of ROC and multivariable logistic regression analyses to evaluate the clinical relevance of XRP-1.

This was a single-center study with a relatively modest sample size. Future multicenter studies incorporating longitudinal measurements and multivariate modeling are warranted to clarify the independent diagnostic and prognostic value of XRP-1.

Although experimental data suggests a potential interaction between XRP-1 and counter-regulatory hormones such as glucagon and cortisol, these hormones were not measured in the present study. Therefore, mechanistic inferences remain speculative. Future studies incorporating concurrent hormonal profiling may clarify the biological pathway linking XRP-1 to insulin resistance in GDM.

Furthermore, formal matching for demographic and metabolic variables was not performed, which may have contributed to baseline differences between groups.

This study evaluated serum XRP-1 levels within the standard GDM screening window (24–28 weeks of gestation) rather than across all trimesters. Therefore, trimester-specific variations in XRP-1 levels were not assessed. Future longitudinal studies evaluating XRP-1 dynamics throughout pregnancy may provide additional insight into its potential role as a biomarker of gestational dysglycemia.

Despite these limitations, our findings suggest that XRP-1 may represent a biologically plausible and clinically relevant biomarker associated with gestational dysglycemia. Further investigation is required to determine whether XRP-1 provides clinically meaningful additional value beyond established clinical and biochemical markers.

## 5. Conclusions

Serum Xenopsin-Related Peptide-1 concentrations were significantly higher in women with gestational diabetes mellitus than in healthy pregnant controls and remained independently associated with GDM after adjustment for established clinical risk factors. Although serum XRP-1 demonstrated only moderate diagnostic accuracy, these findings support its potential role as a complementary biomarker reflecting metabolic dysregulation in GDM rather than as a replacement for established diagnostic methods. Future large-scale, multicenter prospective studies are warranted to validate these findings, investigate the underlying biological mechanisms, and determine whether XRP-1 improves the performance of multimarker prediction models for gestational diabetes mellitus.

## Figures and Tables

**Table 1:** Participants’ obstetric characteristics.

		Control (*n* = 60)	Study (*n* = 60)	*p*
**Age (years)**		27.00 (24.00–30.00)	32.50 (27.75–34.00)	<0.001 *
**BMI (kg/m^2^)**		27.84 (24.24–33.19)	32.72 (28.54–34.16)	<0.001 *
**Weight gained during pregnancy (kg)**		7.52 ± 2.74	12.88 ± 4.60	<0.001 *
**Gravidity**		2.00 (1.00–3.00)	2.50 (1.00–4.00)	0.005 *
**Parity**		0 (0–1)	1 (0–4)	0.023 *
**Curettage history**		0 (0–0)	0 (0–1)	0.251
**Ectopic pregnancy history**		0 (0–0)	0 (0–0)	0.156
**Presentation (*n*, %)**	Vertex	56 (93.3)	55 (1.7%)	0.376
	Breech	4 (6.7)	3 (5.0%)	
	Transverse	0	2 (3.3%)	
**Type of delivery (*n*, %)**	VD	21 (35%)	10 (16.7%)	0.040 *
	C/S	39 (65%)	50 (83.3%)	
**Birth weight (grams)**		3195.54 ± 503.69	3584.21 ± 679.64	<0.013 *
**Fetal gender** **(*n*, %)**	Female	26 (43.3%)	27 (45.0%	0.879
	Male	34 (56.7%)	33 (55.0%)	
**APGAR scores**	1st min.	8.7 ± 0.7	8.4 ± 0.9	0.185
	5th min.	9.9 ± 0.6	9.6 ± 1.1	0.159
**NICU admission rate (*n*, %)**		10 (16.67%)	25 (41.67%)	0.036 *
**First trimester screening** **(*n*, %)**	Low risk	17 (28.3%)	14 (23.3%)	0.142
	High risk	1 (1.7%)	6 (10.0%)	
	None	42 (70.0%)	40 (66.7%)	
**Second trimester screening** **(*n*, %)**	Low risk	44 (73.3%)	38 (63.3%)	0.476
	High risk	1 (1.7%)	2 (3.3%)	
	None	15 (25%)	20 (33.3%)	
**Detailed fetal anatomic screening** **(*n*, %)**	Low risk	10 (16.7%)	13 (21.7%)	0.784
	High risk	1 (1.7%)	1 (1.7%)	
	None	49 (81.7%)	46 (76.7%)	

* Statistically significant. BMI; body mass index, VD; vaginal delivery, C/S; caesarean section, NICU; neonatal intensive care unit.

**Table 2:** Oral glucose tolerance test results of the participants and the treatment they received.

		Control (*n* = 60)	Study (*n* = 60)	*p*
**FBG (mg/dL)**		79.83 ± 14.17	98.40 ± 17.18	<0.001 *
**50 g OGTT 1st hour (mg/dL)**		113.47 ± 25.94	139.64 ± 73.03	0.011 *
**75 g OGTT fasting (mg/dL)**			95.08 ± 3.39	
**75 g OGTT 1st hour (mg/dL)**			184.42 ± 13.99	
**75 g OGTT 2nd hour (mg/dL)**			146.01 ± 15.13	
**100 g OGTT fasting (mg/dL)**		84.14 ± 3.80	102.76 ± 17.64	<0.001 *
**100 g OGTT 1st hour (mg/dL)**		155.14 ± 15.22	186.99 ± 19.43	<0.001 *
**100 g OGTT 2nd hour (mg/dL)**		130.14 ± 10.38	156.76 ± 17.40	<0.001 *
**100 g OGTT 3rd hour (mg/dL)**		110.57 ± 26.46	137.71 ± 17.51	0.003 *
**GDM treatment** **(*n*, %)**	Diet		42 (70.0%)	
	Insulin		18 (30.0%)	

* Statistically significant. GDM; Gestational Diabetes Mellitus, FBG; Fasting blood glucose, OGTT; Oral glucose tolerance test.

**Table 3:** Fetal biometry results of the participants at the time of admission.

		Control (Mean ± SD) (*n* = 60)	GDM (Mean ± SD) (*n* = 60)	*p*
**BPD (mm)**		74.77 ± 10.45	80.32 ± 11.08	<0.001 *
**AC (mm)**		257.54 ± 44.39	292.16 ± 52.99	<0.001 *
**FL (mm)**		55.91 ± 9.78	61.81 ± 10.06	<0.001 *
**AFI** **(*n*, %)**	Normal	56 (93.3%)	43 (71.7%)	0.004 *
	Oligohydramnios	0	2 (3.3%)	
	Polyhydramnios	4 (6.7%)	15 (25%)	
**EFW (estimated fetal weight) at study admission**		1513.37 ± 784.55	2262.53 ± 975.23	<0.001 *

* Statistically significant. BPD; Biparietal Diameter, AC; Abdominal Circumference, FL; Femur Length, EFW; Estimated Fetal Weight.

**Table 4:** Comparison of the laboratory parameters of the participants.

	Control (*n* = 60)	Study (*n* = 60)	*p*
**Hemoglobin (g/dL)**	11.20 (10.55–12.10)	11.50 (10.80–12.12)	0.213
**Hematocrit (%)**	33.55 (31.35–35.55)	34.80 (32.77–36.58)	0.025
**Leukocyte count (10^3^/µL)**	10.90 (9.75–12.65)	10.75 (9.00–12.53)	0.458
**Platelet count (10^3^/µL)**	244.00 (207.25–297.00)	239.00 (188.50–296.75)	0.408
**Neutrophil count (10^3^/µL)**	8.21 (6.55–9.49)	7.77 (6.79–9.08)	0.665
**Lymphocyte count (10^3^/µL)**	1.89 (1.68–2.28)	2.12 (1.64–2.45)	0.219
**NLR**	3.96 (3.35–5.11)	3.78 (3.08–4.51)	0.143
**PLR**	135.9 ± 44.04	122.58 ± 40.9	0.086
**Pan-immune inflammatory index**	725.04 ± 175.37	809.97 ± 163.96	0.324
**ALT (IU/L)**	13.00 (10.00–15.25)	11.00 (8.75–16.00)	0.201
**AST (IU/L)**	15.50 (13.00–18.00)	15.00 (12.00–18.50)	0.521
**BUN (mg/dL)**	7.00 (6.00–8.00)	8.00 (7.00–9.00)	0.011
**Creatinine (mg/dL)**	0.62 (0.58–0.66)	0.65 (0.60–0.72)	0.026
**CRP (mg/L)**	3.00 (2.00–5.25)	5.50 (4.00–8.00)	<0.001 *
**Na (mmol/L)**	136.82 ± 3.16	135.47 ± 2.93	0.568
**K (mmol/L)**	3.81 ± 0.38	3.95 ± 0.40	0.206
**Cl (mmol/L)**	100.77 ± 19.55	103.16 ± 21.13	0.428
**TSH (µIU/mL)**	1.84 ± 0.51	2.08 ± 0.61	0.309
**fT4 (Free thyroxine) (ng/dL)**	0.65 ± 0.11	0.64 ± 0.10	0.597
**fT3 (free triiodothyronine) (pg/dL)**	3.26 ± 0.29	3.38 ± 0.31	0.104
**Glucose (mg/dL)**	80.00 (72.75–88.25)	88.00 (81.75–107.25)	<0.001 *
**Urine glucose (semiquantitative)**	0.00 (0.00–0.00)	0.00 (0.00–0.00)	0.053
**Urine protein (semiquantitative)**	0.05 ± 0.01	0.05 ± 0.01	0.655
**Urine ketones (semiquantitative)**	0.00 (0.00–0.00)	0.00 (0.00–0.00)	1.000
**Urine leukocytes (semiquantitative)**	1.50 (0.00–4.00)	2.00 (0.00–9.00)	0.073
**HbA1c (%)**	5.08 ± 0.35	6.13 ± 0.71	<0.001 *
**XRP-1**	2.87 ± 0.46	3.45 ± 0.47	0.003 *

* Statistically significant. NLR; neutrophil-lymphocyte ratio, PLR; platelet-lymphocyte ratio, ALT; alanine aminotransferase, AST; aspartate ştığıaminotransferase, BUN; blood urea nitrogen, CRP; c-reactive protein, HbA1c; glycated hemoglobin, XRP-1; Xenopsin-Related Peptide-1.

**Table 5:** Pregnancy complications in women with GDM.

		Control (*n* = 60)	GDM (*n* = 60)
**Pregnancy Complications** **(*n*, %)**	Preeclampsia		8 (13.3%)
	Preterm birth		11 (18.3%)
	Polyhydramnios Oligohydramniosis		16 (26.7%)
	FGR		3 (5.0%)
	Placenta previa		5 (8.3%)
	Gestational Cholestasis		4 (6.7%)

FGR; fetal growth restriction.

**Table 6:** Sensitivity, specificity, positive and negative predictive value of Xenopsin-Related Peptide-1.

Parameter	AUC	*p*-Value	Lower 95% CI	Upper 95% CI	Sensitivity (%)	Specificity (%)	PPV (%)	NPV (%)
XRP-1	0.658	0.004	0.560	0.750	63.30%	61.70%	62.10%	62.90%

AUC: Area under curve, CI: confidence interval, PPV: positive predictive value, NPV: negative predictive value.

## Data Availability

Data supporting the findings of this study are available from the corresponding author upon reasonable request.

## Abbreviations

The following abbreviations are used in this manuscript:

AC	Abdominal Circumference
AFI	Amniotic Fluid Index
ALT	Alanine Aminotransferase
AST	Aspartate Aminotransferase
AUC	Area Under the Curve
BMI	Body Mass Index
BPD	Biparietal Diameter
BUN	Blood Urea Nitrogen
CI	Confidence Interval
CRP	C-Reactive Protein
ELISA	Enzyme-Linked Immunosorbent Assay
EFW	Estimated Fetal Weight
FL	Femur Length
FBG	Fasting Blood Glucose
FGR	Fetal Growth Restriction
GDM	Gestational Diabetes Mellitus
HbA1c	Glycated Hemoglobin
IQR	Interquartile Range
NICU	Neonatal Intensive Care Unit
NLR	Neutrophil-to-Lymphocyte Ratio
NPV	Negative Predictive Value
OGTT	Oral Glucose Tolerance Test
OR	Odds Ratio
PLR	Platelet-to-Lymphocyte Ratio
PPV	Positive Predictive Value
ROC	Receiver Operating Characteristic
SD	Standard Deviation
TSH	Thyroid-Stimulating Hormone
XRP-1	Xenopsin-Related Peptide-1
